# Impact of Adjunct Testosterone on Cancer-Related Fatigue: An Ancillary Analysis from a Controlled Randomized Trial

**DOI:** 10.3390/curroncol29110658

**Published:** 2022-11-01

**Authors:** Kristen A. McGovern, William J. Durham, Traver J. Wright, E. Lichar Dillon, Kathleen M. Randolph, Christopher P. Danesi, Randall J. Urban, Melinda Sheffield-Moore

**Affiliations:** Department of Internal Medicine, The University of Texas Medical Branch (UTMB), 301 University Blvd., Galveston, TX 77555, USA

**Keywords:** cancer, CRF, cytokines, fatigue, mood, quality of life, testosterone

## Abstract

Many cancer patients undergoing treatment experience cancer-related fatigue (CRF). Inflammatory markers are correlated with CRF but are not routinely targeted for treatment. We previously demonstrated in an NIH-funded placebo-controlled, double-blind, randomized clinical trial (NCT00878995, closed to follow-up) that seven weekly injections of 100 mg adjunct testosterone preserved lean body mass in cancer patients undergoing standard-of-care treatment in a hospital setting. Because testosterone therapy can reduce circulating proinflammatory cytokines, we conducted an ancillary analysis to determine if this testosterone treatment reduced inflammatory burden and improved CRF symptoms and health-related quality of life. Randomization was computer-generated and managed by the pharmacy, which dispensed testosterone and placebo in opaque syringes to the administering study personnel. A total of 24 patients were randomized (14 placebo, 10 testosterone), and 21 were included in the primary analysis (11 placebo, 10 testosterone). Testosterone therapy did not ameliorate CRF symptoms (placebo to testosterone difference in predicted mean multidimensional fatigue symptom inventory scores: −5.6, 95% CI: −24.6 to 13.3), improve inflammatory markers, or preserve health-related quality of life and functional measures of performance in late-stage cancer patients.

## 1. Introduction

Cancer and associated treatments render many patients with fatigue, marked by a general perception of weariness, tiredness, and/or lack of energy. This cancer-related fatigue (CRF) is typically reported by cancer patients as their most distressing symptom [1,2,3,4,5]. Nearly all patients experience moderate to severe CRF at some point during therapy [1,2], and CRF severity is predictive of survival [6]. Because CRF is a significant implicit component in the clinical equation used to derive a cancer patient’s performance status [6,7,8,9], it is a critical determinant of treatment course [10].

Despite the high incidence and prominence of CRF symptomatology, its etiology remains poorly defined [2,11,12]. Proposed factors mediating CRF include pro-inflammatory signaling [2,11,13,14,15,16], loss of skeletal muscle [17], circadian rhythm disruption [18], and hypothalamic-pituitary-adrenal axis disruption [19], but experimental support and mechanistic details for these and other factors remains limited [19].

Given the poor understanding of the etiology of CRF, the lack of treatments for this symptom is perhaps unsurprising. While stimulants have been the most commonly employed pharmacological approach to treat CRF, many studies have reported no improvement, with increased incidence of adverse effects in some cases [20,21,22,23,24,25,26,27,28,29,30,31]. Many cancer patients receive glucocorticoid therapy to ameliorate treatment-related side effects, but with limited efficacy [32,33]. Regardless, glucocorticoid therapy is a short-term option at best for cancer-related fatigue, because prolonged use induces insulin resistance, increased adiposity [34,35,36], muscle wasting [37], brain atrophy, and diminished neurogenesis [38,39]. Chronic exercise training is the most effective treatment for CRF [2,8,40,41,42,43,44,45], suggesting that increased muscle activity triggers cellular and metabolic adaptations that counteract factors responsible for CRF. Although diet has received little attention in CRF, diet quality has been associated with reduced fatigue in a cross-sectional study of breast cancer survivors following primary treatment [46]. This may be due to dietary effects on the gut microbiome [47,48], which has been linked to fatigue in cancer patients and survivors [49,50].

Adding further complexity to the etiology of CRF, cancer patients also exhibit a wasting phenotype called cancer cachexia, which is responsible for roughly one in five cancer deaths [51]. Cancer cachexia is characterized by persistent loss of skeletal muscle mass resulting in worsening functional impairment which cannot be restored via standard nutritional support [52]. CRF and cachexia share etiological factors including anorexia, inflammation, and inactivity [53], and may function synergistically to the detriment of cancer patients. 

By exploring potential treatments to ameliorate cancer-related symptoms common to both cachexia and fatigue, we aim to improve patient quality of life, expand treatment options, and increase time of survival. To that end, we previously demonstrated in a double-blind, placebo-controlled trial that weekly testosterone therapy preserved lean body mass in cervical and head and neck cancer patients with advanced or recurrent squamous cell carcinoma [54]. In addition to its anti-catabolic properties, testosterone has been demonstrated to have anti-inflammatory effects. Testosterone levels negatively correlate with inflammatory markers [55] and testosterone therapy has been associated with decreases in serum levels of proinflammatory cytokines [56,57,58]. Because inflammatory markers and cachexia are associated with fatigue [13,59], we hypothesized that adjunct testosterone would improve fatigue symptoms and health-related quality of life through the mechanism of reducing inflammatory burden. In this planned ancillary analysis we report whether weekly testosterone therapy improves inflammatory markers, CRF symptom severity, and health-related quality of life. 

## 2. Materials and Methods

We conducted a randomized, double-blind, placebo-controlled clinical trial (National Clinical Trials number NCT00878995) which was approved by the Institutional Review Board at the University of Texas Medical Branch and carried out in conformity with the Declaration of Helsinki. All subjects provided written informed consent before enrollment and received standard-of-care therapy without restriction by the study team. The previously reported primary endpoint was the percent change in lean body mass during approximately 7 weeks of experimental intervention in cancer patients with advanced or recurrent squamous cell carcinoma of the cervix or head and neck [54]. Power analysis for this primary outcome indicated that a sample size of 6–12 patients per group would provide power from 0.87 to >0.99 to detect changes. Previously reported secondary outcomes were lean body mass, fat mass, energy expenditure, peak isometric strength, peak isokinetic strength, physical activity, quality of life, 1-year survival, energy intake, and select laboratory values. The experimental intervention consisted of weekly adjunct administration of either testosterone (100 mg) or placebo (sterile saline) injected intramuscularly during standard-of-care cancer treatment. Details of the study protocol, including timing of sampling, testosterone intervention paradigm, and patient eligibility, were presented previously, and are briefly discussed here.

Patients were eligible for the study if they reported unintentional loss of at least 5% of body mass in the preceding 12 months and were either (1) 18 to 65 years of age and diagnosed with advanced or recurrent squamous cell carcinoma of the cervix (stages IIB, IIIA, IIIB, and IV), or (2) 18 to 75 years of age and diagnosed with advanced or recurrent head and neck squamous cell carcinoma. Eligibility requirements also included being ambulatory with some activity (Eastern Cooperative Oncology Group score of 0–1) and scoring at least 23 out of 30 on the Mini Mental State Examination [2], which was required to sign the study consent form.

**Figure 1:**
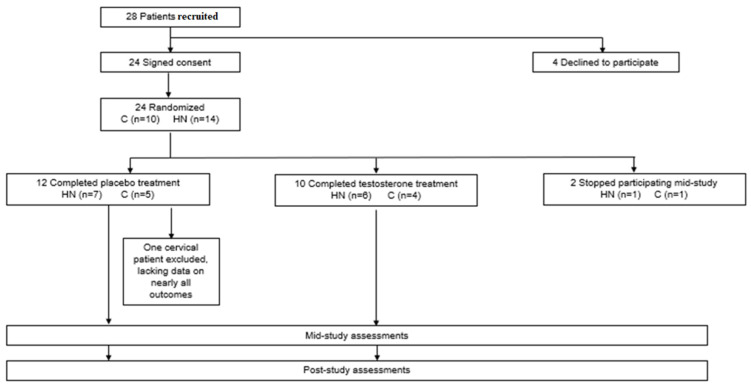
History of study participants from enrollment through post-study assessments.

Eligible participants were recruited from April 2009 to April 2014. A total of 28 patients were screened for participation. Of these, 24 provided consent and were randomized using a computerized random number generator by the institutional research pharmacy in blocks of 3 to receive weekly intramuscular injections of either 100 mg of testosterone (*N* = 10) or placebo (*N* = 14). Recruitment was ended once 24 patients were randomized, in accordance with the sample size indicated by the previously mentioned power analysis. The UTMB Investigational Drug Service pharmacy managed study group assignment and dispensed testosterone and placebo in opaque syringes to the administering study personnel, who were blinded. Two patients stopped participating mid-study and one patient that was included in the initial report was excluded due to missing data for almost all measurements (Figure 1). One female patient in the testosterone group met criteria for inclusion in this analysis, although she was excluded from the original report due to multiple primary tumors. Patients were followed up via monthly phone call for one year following the intervention. The final participant completed the intervention in June 2014; follow-up for all patients was completed by the end of June 2015. Adverse events were previously reported [54]; no adverse events were reported for the additional participant included in this analysis.

Patient data were collected at the University of Texas Medical Branch Institute for Translational Sciences Clinical Research Center in Galveston, TX. Standard-of-care and treatment schedule varied by participant, which was taken into consideration by the study protocol. Mean study duration was 47.8 ± 10 days in the placebo group and 47.0 ± 7.7 days in the testosterone group, with mid-treatment measures taken within 4.6 ± 4.1 days of each subject’s study midpoint, and post-treatment measures taken within 1.1 ± 3.7 days of the final experimental injection. 

### 2.1. Blood Sampling for Inflammatory Markers and Amino Acids

Serum cytokine levels were measured pre- and post-intervention using the MILLIPLEX MAP Human High Sensitivity T-Cell Panel Premixed 13-plex—Immunology Multiplex Assay, following the manufacturer’s instructions. Concentrations of granulocyte-macrophage colony-stimulating factor (GM-CSF), interferon-gamma (IFN-γ), interleukin (IL)-1β, IL-2, IL-4, IL-5, IL-6, IL-7, IL-8, IL-10, IL-12, IL-13, and tumor necrosis factor (TNF-α) were determined. Sample measurements below the limit of detection were set to 0.5 * the detection limit. Serum amino acid concentrations were measured pre- and post-intervention using a Hitachi L8800 amino acid analyzer according to manufacturer guidelines.

### 2.2. Questionnaires to Assess Fatigue, Mood, and Quality of Life

#### 2.2.1. Fatigue

Cancer-related fatigue was assessed using the MD Anderson Brief Fatigue Inventory (BFI) [7], a visual analog scale (VAS) for fatigue, and the Multidimensional Fatigue Symptom Inventory—Short Form [60] (MFSI). The BFI is a 9-item questionnaire assessing both the degree of cancer-related (perceptual) fatigue and how it interferes with various aspects of patients’ lives. The BFI assesses cancer-related fatigue at the present moment (“right now”), over the past 24 h, and over the past 7 days. The VAS for fatigue consists of a 10 cm horizontal line with the left extreme labeled “No fatigue” and the right extreme labeled “Extreme fatigue”. Patients were instructed to mark a vertical line at the point along the horizontal line that corresponded to their overall level of fatigue. The MFSI-SF consists of 30 statements for which cancer patients provide a self-rating of the extent of agreement. The items are rated on a 5-point scale, with 0 indicating the statement is not at all true with respect to their lives over the last 7 days and 4 indicating the statement is very true with respect to their lives over the last 7 days. The individual item responses are used to calculate empirically-derived subscales assessing general, physical, emotional, and mental fatigue, as well as vigor. Minimal clinically important difference criteria established in Asian breast cancer patients were used to determine whether subjects experienced clinically meaningful deterioration of fatigue between measurements [61]. Normative data for MFSI subscales is available separately for African American, Caucasian, and Hispanic adults [62,63], but there is no pooled normative data available. We did not have a sufficient sample size to conduct statistical comparisons using each of the normative data sets. In the interest of conducting a statistical comparison, and because the majority (>70%) of study participants were Caucasian, pre-treatment MFSI subscale scores were compared to normative data for Caucasian adults [62]. 

#### 2.2.2. Mood

Mood was assessed using the Profile of Mood States—Short Form [64] (POMS), which produces an overall score of mood disturbance as well as six subscales: tension-anxiety, depression–dejection, anger–hostility, vigor–activity, fatigue–inertia, and confusion–bewilderment. Pre-treatment POMS subscale scores of study participants were compared to normative data for adults > 25 years of age [65].

#### 2.2.3. Health-Related Quality of Life

Health-related quality of life (QOL) was measured using the RAND 36-Item Health Survey 1.0 questionnaire [66] (SF-36); responses are used to construct 8 scales: physical functioning, emotional well-being, role limitations due to physical health, role limitations due to emotional problems, social functioning, energy/fatigue, pain, and general health. Pre-treatment SF-36 subscale scores of study participants were compared to normative data for adults [67]. The MD Anderson Symptom Inventory [3] (MDASI) was also administered, with responses used to construct scores for symptom severity and interference. Nausea was measured using a VAS format similar to the VAS for fatigue. Functional Assessment of Cancer Therapy [68] (FACT-G) questionnaire scores were previously reported [54], and are included here in supporting correlative analyses and characterizations.

The MFSI, SF-36, and POMS questionnaires were administered at the pre-, mid-, and post-treatment time points. Missing responses on completed questionnaires were minimal (<1%) and were imputed for the MFSI and POMS using the average of the participant’s remaining responses for the applicable subscale for a maximum of one question per subscale [69]. The BFI, MDASI, and VAS questionnaires were administered daily over the course of the intervention and averaged by week; weekly scores were included in analyses if a minimum of one questionnaire was completed for that week.

### 2.3. Statistical Analyses

Statistical analyses were conducted using GraphPad Prism (Version 9.4.1; GraphPad Software, La Jolla, CA, USA) and R (Version 4.2.1); the threshold for statistical significance was set at *p* < 0.05. Groupwise comparisons of continuous and discrete demographics variables were analyzed using unpaired Welch’s *t*-tests and Fisher’s exact test, respectively. Average percent changes from baseline to post-treatment were determined for serum cytokine concentrations and compared between the testosterone and placebo groups using unpaired Welch’s *t*-tests; *p*-values were adjusted for multiple testing using a Holm–Šídák correction. Only patients with both baseline and post-treatment serum cytokine measurements were included in this analysis. Linear mixed effects models (with Geisser–Greenhouse correction) were used to test the main effects of treatment (placebo vs. testosterone) and time point (pre, mid, and post), as well as the interaction effect of treatment by time point on questionnaire scores and subscales; *p*-values were adjusted for multiple testing using a Bonferroni correction. Linear mixed effects models are maximum likelihood approaches suitable for assessing effects when data are missing. Data for all patients were included in these analyses; missing values for each model are reported in the Supplementary Materials. Pre-treatment scores for the MFSI, SF-36, and POMS questionnaires were compared to normative data from previous studies in the literature using unpaired Welch’s *t*-tests; *p*-values were adjusted for multiple testing using a Holm–Šídák correction.

In an exploratory analysis, data were pooled and a Spearman’s rank correlation heat map was created to identify correlations among baseline serum cytokine levels and questionnaire scores. Correlation coefficients exceeding an absolute value of 0.7 with *p* < 0.05 are presented in the text. Due to the exploratory nature of this analysis, *p*-values for correlations were not corrected for multiple testing.

## 3. Results

### 3.1. Baseline Demographics

Baseline demographics, including age, body mass, and BMI, as well as the distributions of sex, race, cancer type and tumor stage, were similar between placebo and testosterone groups (Table 1). Kaplan–Meier survival curves were not significantly different between groups, and were similar to previously reported curves with a slightly different patient set [54] (Figure S1).

### 3.2. Correlations of Baseline Cytokine Levels with Questionnaire Scores

**Figure 2:**
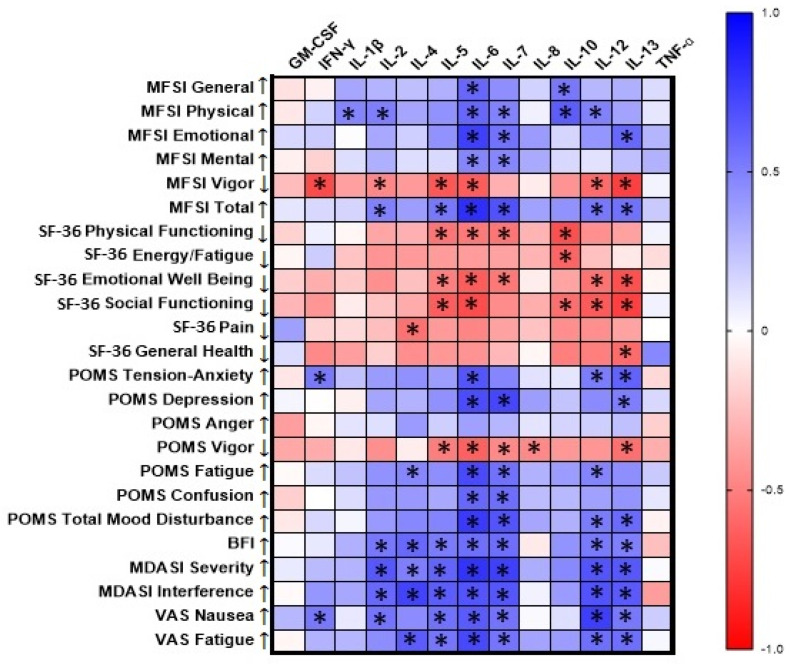
Correlations among baseline questionnaire scores and serum cytokine levels in cancer patients. Statistically significant correlations are denoted with an asterisk. Downward arrows indicate that lower scores are undesirable and indicate greater symptom severity; upward arrows indicate that higher scores are undesirable and indicate greater symptom severity. MFSI—Multidimensional Fatigue Symptom Inventory—Short Form, SF-36—RAND 36-Item Health Survey 1.0, POMS—Profile of Mood States—Short Form, BFI—Brief Fatigue Inventory, MDASI—MD Anderson Symptom Inventory, VAS—Visual Analog Scale.

Baseline cytokine levels were significantly correlated with questionnaire scores evaluating patient self-assessment of fatigue, mood, and symptom severity (Figure 2). Significant correlations with |r| ≥ 0.7 are listed. IFN-γ was correlated with the MFSI Vigor subscale (r = −0.71, *p* < 0.001, *n* = 18). IL-4 was correlated with MDASI Interference (r = 0.74, *p* < 0.001, *n* = 17). IL-6 was correlated with the MFSI Emotional subscale (r = 0.75, *p* < 0.001, *n* = 18) and Total score (r = 0.82, *p* < 0.001, *n* = 18), the SF-36 Social Functioning scale (r = −0.71, *p* = 0.002, *n* = 16), the POMS Depression (r = 0.70, *p* = 0.001, *n* = 18) and Fatigue (r = 0.71, *p* < 0.001, *n* = 18) subscales and Total Mood Disturbance score (r = 0.77, *p* < 0.001, *n* = 17), MDASI Severity (r = 0.80, *p* < 0.001, *n* = 17), and VAS Fatigue (r = 0.71, *p* = 0.002, *n* = 16). IL-7 was correlated with the POMS Depression subscale (r = 0.72, *p* < 0.001, *n* = 18) and MDASI Severity (r = 0.75, *p* < 0.001, *n* = 17). IL-12 was correlated with VAS Nausea (r = 0.75, *p* < 0.001, *n* = 17). IL-13 was correlated with the MFSI Vigor subscale (r = −0.75, *p* < 0.001, *n* = 18) and the SF-36 Social Functioning subscale (r = −0.75, *p* = 0.001, *n* = 16).

### 3.3. Comparisons of Baseline Questionnaire Scores to Normative Data

Baseline MFSI subscale scores of study participants were not significantly different from previously published normative data (Table 2). Baseline POMS Vigor scores for cervical or head and neck cancer patients were significantly lower than those of normative subjects, indicating greater symptom severity. Other POMS subscale and POMS Total Mood Disturbance scores were not significantly different from those of normative subjects (Table 2). Baseline measures of SF-36 Physical Functioning, Role Limitations Due to Physical Health, and Social Functioning subscales for cervical or head and neck cancer patients were significantly lower than those of normative subjects, indicating greater symptom severity. Other SF-36 subscale scores were not significantly different from those of normative subjects (Table 2).

### 3.4. Serum Cytokine Levels

**Figure 3:**
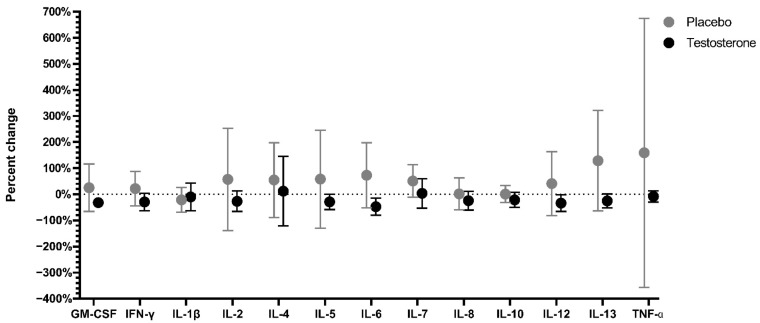
Percent change (average ± SD) in serum cytokine concentrations from baseline to post-treatment for cancer patients receiving testosterone (*n* = 7) or placebo *(n* = 11). There were no significant differences between groups after correcting for multiple testing. Abbreviations: GM-CSF—granulocyte-macrophage colony-stimulating factor, IFN-γ—interferon-gamma, IL—interleukin, tumor necrosis factor—TNF-α.

Serum cytokine levels were highly variable in the placebo and testosterone groups at baseline and post-treatment (Table S1). There were no significant differences between groups in percent changes of serum cytokine concentrations from baseline to post-treatment (Figure 3, Table S2).

### 3.5. Serum Amino Acid Levels

Baseline and post-treatment serum amino acid concentrations are presented in Table S4. There were no differences in percent change of serum amino acid and associated metabolite concentrations from baseline to post-treatment between the placebo (*n* = 9) and testosterone (*n* = 9) groups (Figure S2, Table S5).

### 3.6. Effects of Treatment and Time Point on Fatigue, Mood, and Quality of Life

#### 3.6.1. Fatigue

**Figure 4:**
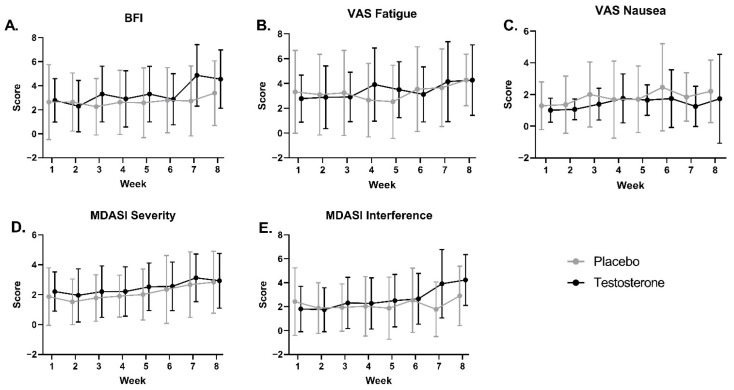
Daily questionnaire scores averaged by week for cancer patients receiving testosterone or placebo. Average (±SD) weekly scores for (**A**) Brief Fatigue Inventory—BFI, (**B**) visual analog scale for fatigue—VAS Fatigue, (**C**) visual analog scale for nausea—VAS Nausea, (**D**) MD Anderson Symptom Inventory severity—MDASI Severity, and (**E**) MD Anderson Symptom Inventory interference—MDASI Interference. There were no significant differences between groups or in scores over time for either group. Higher scores on presented questionnaires are undesirable and indicate greater symptom severity.

**Figure 5:**
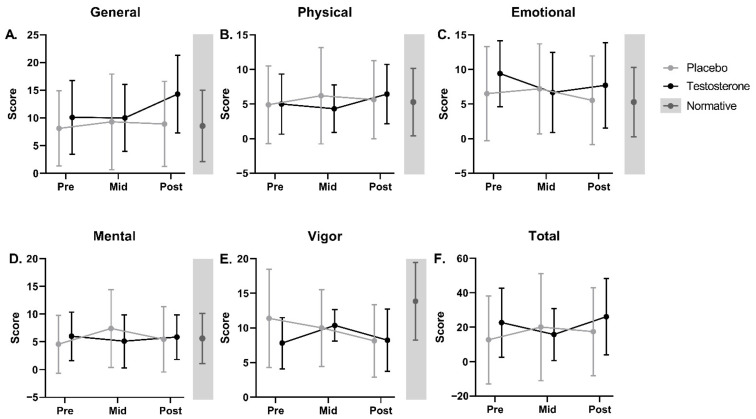
Multidimensional Fatigue Symptom Inventory—Short Form (MFSI) subscale and total scores at pre, mid, and post for cancer patients receiving testosterone or placebo. Average (±SD) scores for (**A**) General Fatigue, (**B**) Physical Fatigue, (**C**) Emotional Fatigue, (**D**) Mental Fatigue, and (**E**) Vigor subscales, as well as (**F**) Total score. Average (±SD) normative scores [62], as available, are displayed in gray sidebars to the right of each graph. There were no significant differences between groups or in scores over time for either group. Higher scores on presented questionnaires are undesirable and indicate greater symptom severity, with the exception of Vigor (**E**), for which lower scores are undesirable and indicate greater symptom severity.

The linear mixed effects model results demonstrated no significant main effects of treatment or time and no significant interaction effect of treatment by time for weekly averaged BFI or VAS Fatigue scores over the 7-week intervention (Figure 4A,B, Table S3). There were no significant differences in pre-, mid-, and post-treatment MFSI total scores or associated subscales (Vigor, as well as General, Physical, Emotional, and Mental fatigue) between treatment groups or longitudinally within groups (Figure 5, Table S3). Based on previously established criteria for minimal clinically important difference (increase in MFSI total score > 10.79), 40% of the placebo group and 37.5% of the testosterone group had a clinically significant increase in perceived fatigue between the pre- and post-treatment time points [61].

#### 3.6.2. Mood

**Figure 6:**
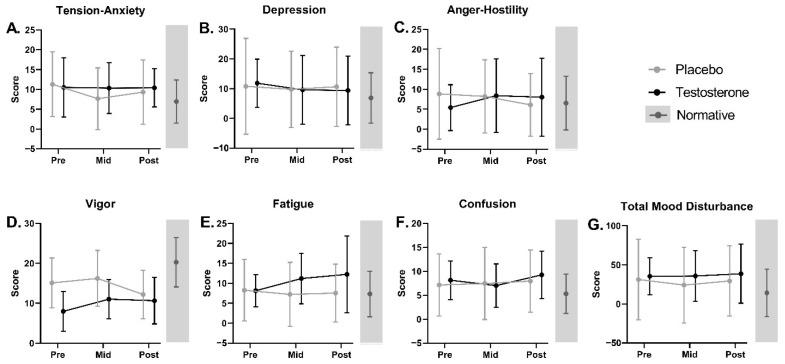
Profile of Mood States—Short Form (POMS) subscale and total scores at pre, mid, and post for cancer patients receiving testosterone or placebo. Average (±SD) scores for (**A**) Tension–Anxiety, (**B**) Depression, (**C**) Anger–Hostility, (**D**) Vigor, (**E**) Fatigue, and (**F**) Confusion subscales, as well as (**G**) Total Mood Disturbance score. Average (±SD) normative scores [65], as available, are displayed in gray sidebars to the right of each graph. There were no significant differences between groups or in scores over time for either group after adjusting for multiple testing. Higher scores on presented questionnaires are undesirable and indicate greater symptom severity, with the exception of Vigor (**D**), for which lower scores are undesirable and indicate greater symptom severity.

There were no significant main effects of treatment or time and no significant treatment by time interaction effect for POMS total scores and subscales (including Tension–Anxiety, Depression, Anger–Hostility, Vigor, Fatigue, Confusion, and Total Mood Disturbance) over the 7-week study (Figure 6, Table S3).

#### 3.6.3. Health-Related Quality of Life

**Figure 7:**
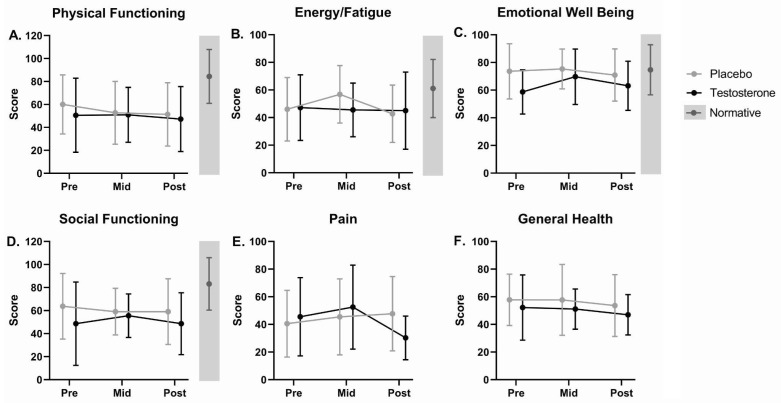
RAND 36-Item Health Survey 1.0 (SF-36) scale scores at pre, mid, and post for cancer patients receiving testosterone or placebo. Average (±SD) scores for (**A**) Physical Functioning, (**B**) Energy/Fatigue, (**C**) Emotional Well Being, (**D**) Social Functioning, (**E**) Pain, and (**F**) General Health scales. Average (±SD) normative scores [67], as available, are displayed in gray sidebars to the right of each graph. There were no significant differences between groups or in scores over time for either group. Lower scores on presented questionnaires are undesirable and indicate greater symptom severity.

There were no significant differences in pre-, mid-, and post-treatment SF-36 subscales, including Physical Functioning, Energy/Fatigue, Emotional Well Being, Social Functioning, Pain, and General Health, between groups or longitudinally within groups over the 7-week intervention (Figure 7, Table S3). There were no significant main effects of treatment or time and no significant interaction effect of time by treatment for weekly averaged VAS Nausea scores (Figure 4C, Table S3) or MDASI Severity or Interference scales over the 7-week intervention (Figure 4D,E, Table S3).

## 4. Discussion

Previously, we demonstrated that adjunct testosterone preserved lean body mass and improved SPPB scores, but did not alter peak power or torque generated by leg extension in cervical or head and neck cancer patients undergoing standard-of-care treatment [54]. Here, we show that despite its anti-catabolic effects, seven weeks of adjunct testosterone did not improve inflammatory markers and did not ameliorate CRF in these patients.

Care should be taken to distinguish between peripheral and central fatigue. While peripheral fatigue is associated with physical exhaustion and occurs within the muscle [70,71,72,73,74], central fatigue originates in the central nervous system and encompasses the cognitive element of mental fatigue [75,76]. Previous research indicates that CRF is central in origin [70,77]. Only a portion of cancer patients develop CRF, which is presumably caused by an aggregation of physical and psychosocial risk factors, with some individuals likely more susceptible than others [11].

In this study, baseline objective measures (i.e., cytokine levels) were highly correlated with subjective measures of fatigue, mood, health-related quality of life, and symptom severity and interference. However, it is important to note that the *p*-values for this exploratory analysis were not adjusted for multiple testing. The cytokine that was most highly correlated with fatigue and neuropsychological measures at baseline in the cervical and head and neck cancer patients in this study was IL-6. Serum IL-6 has previously been observed to be elevated in cancer patients compared to controls [78] and has been linked to tumor burden and fatigue in cancer patients, both pre- and post-treatment [15,79,80]. High serum IL-6 levels can indicate poor prognosis in breast cancer patients [81], and has also been correlated with fatigue in other conditions [82,83]. Low doses of IL-6 administered to healthy male volunteers resulted in higher reported fatigue, diminished ability to concentrate, and reduced REM sleep [84].

Cytokine levels did not change significantly over the course of treatment. Although testosterone has demonstrated anti-inflammatory effects via reduction of cytokine concentrations in previous studies [56,57,58], it did not suppress inflammatory cytokine burden in this study. This is likely due to the combined effects of cancer and its treatment on cytokine levels. Pro-inflammatory cytokines can be secreted in response to the tumor itself or in response to cancer therapy [13,85] and can be secreted from cancer cells, immune cells, or nervous system cells [14]. Any decrease in cytokine secretion due to reduction of tumor mass following chemotherapy or radiation was likely offset by an increase in cytokines due to the treatment. Men and women received the same dose (100 mg) of testosterone in this study [54]. While this is a typical dose to treat hypogonadism in men, it is a hyper-physiological dose for women.

Recent research suggests that the complex of symptoms affecting fatigue, cognition, and mood in cancer patients might be present prior to standard-of-care treatment [86,87,88,89,90,91,92,93,94]. Compared to normative data, cancer patients in this study reported significantly greater symptom severity at baseline for questionnaire subscales corresponding to vigor, physical and social functioning, and role limitations due to physical health following adjustment for multiple testing. Cancer patients in this study also reported greater symptom severity at baseline for questionnaire scores corresponding to emotional fatigue, energy/fatigue, tension–anxiety, and total mood disturbance, although these comparisons were not significant after correcting for multiple testing. Although our previous results indicated that testosterone therapy resulted in clinically meaningful improvements in the FACT-G questionnaire total score and social/family well-being subscale [54], adjunct testosterone did not improve fatigue, mood, health-related quality of life, or symptom severity and interference outcomes reported in this analysis. Not only were fatigue and other measures assessed by questionnaire unaltered by testosterone therapy in this study, they were unchanged over the course of standard-of-care treatment. This might reflect the advanced stage of many of the patients in this trial.

CRF and other complex cancer-related symptom clusters are unlikely to be fully predicted exclusively by individual cytokines or inflammatory markers. CRF has a strong association with cancer-related cognitive impairment (CRCI) [95], another distressing symptom experienced by up to 75% of patients undergoing cancer treatment [96]. CRCI is characterized by deficiencies in various cognitive domains including concentration, attention, and executive function [96]. Possible factors mediating CRCI include activation of microglia, which may block neurogenesis or alter neural impulse time [97], hippocampal shrinkage caused by glucocorticoids [38,39], and effects from chemotherapy such as hippocampal oxidative damage [85] and neuroinflammation, which is linked to cognitive impairment due to alterations in myelin structure and myelination [98]. The absence of standardized assessments for CRCI [99] and limited association between patient-perceived cognitive changes and neurocognitive test results [100] make it difficult to identify, measure, and track CRCI in cancer patients.

Cancer patients also experience alterations to the gut microbiome as a result of both chemotherapy [101,102] and the cancer itself [103]. We have observed a similar constellation of symptoms, including fatigue and cognitive impairment, in some patients with traumatic brain injury, and these patients additionally present with an altered gut microbiome and growth hormone deficiency [104,105]. Comparable symptoms of fatigue, cognitive dysfunction, and gut microbiome changes have been observed in patients with post-acute sequelae of SARS-CoV-2 infection, or “long Covid” [106,107], and chronic fatigue syndrome [108,109], among other disorders [110,111,112]. While one study found no differences in gut microbiome diversity based on level of fatigue [113], two studies did report an association between fatigue and changes to the gut microbiome in cancer patients or survivors [49,50]. Additionally, one of these studies found that patients in the high fatigue group had a greater abundance of taxa linked to inflammation [50], while the other found that relative abundance of certain taxa correlated with neurocognitive capacity in cancer survivors [49].

### Study Limitations

A chief limitation to this study was the small number of study participants. This study was originally powered based on the primary outcome of change in lean body mass following testosterone therapy [54] and was not directly powered for the secondary outcomes presented here. The main outcome in this ancillary analysis was perceptual fatigue as assessed by the MFSI Total Score. There was no trend in fatigue scores observed over the course of the study, so it is unknown whether a difference would have been detected with a larger sample size. The testosterone group scored slightly higher than the placebo group at baseline and the post-treatment timepoint, but slightly lower at the study mid-point. Another limitation was that there was missing outcome data for some of the measures, so individual patients may be included in some analyses but not others.

## 5. Conclusions

Despite preserving lean body mass, testosterone treatment did not reduce inflammatory burden, improve fatigue, or preserve health-related quality of life and functional measures of performance in late-stage cancer patients. This discrepancy suggests that these factors are independent and should be considered individually when devising a clinical treatment plan for patients. Further studies are needed to elucidate mechanisms contributing to the symptom complex of CRF, CRCI, and mood disturbance in cancer patients prior to, during, and following primary and adjuvant therapy. The role of the microbiome in the etiology of these symptoms warrants further investigation.

## Figures and Tables

**Table 1:** Demographics of cervical or head and neck cancer patients receiving 7 weeks of adjunct testosterone or placebo.

		Total	Placebo	Testosterone	
		(*N* = 21)	(*N* = 11)	(*N* = 10)	*p*
Age (y)	Mean ± SD	51.9 ± 10.1	50.1 ± 11.8	53.9 ± 8	0.39
	Range	35–71	35–71	35–61	
					
Body Mass (initial, kg)	Mean ± SD	65.4 ± 22.6	69.4 ± 25.7	60.9 ± 19.1	0.40
	Range	22.6–130.7	44.4–130.7	39–101	
					
BMI (initial)	Mean ± SD	23 ± 6.8	23.9 ± 6.9	22 ± 6.9	0.55
	Range	14.5–40.3	16.3–40.3	14.5–37.6	
					
Days in study	Mean ± SD	47.5 ± 8.8	47.8 ± 10	47 ± 7.7	0.81
					
Sex—no. (%)	Male	10 (47.6)	7 (63.6)	3 (30)	0.20
	Female	11 (52.4)	4 (36.3)	7 (70)	
					
Race—no. (%)	Black	4 (19.1)	2 (18.2)	2 (20)	0.52
	Caucasian	15 (71.4)	7 (63.6)	8 (80)	
	Hispanic	2 (9.5)	2 (18.2)	0 (0)	
					
Tumor Stage—no. (%)	IIB	2 (9.5)	0 (0)	2 (20)	0.71
	III	1 (4.8)	1 (9.1)	0 (0)	
	IIIB	5 (23.8)	3 (27.3)	2 (20)	
	IV	1 (4.8)	1 (9.1)	0 (0)	
	IVA	9 (42.9)	4 (36.4)	5 (50)	
	IVB	3 (14.3)	2 (18.2)	1 (10)	
					
Cancer Type—no. (%)	Cervical	8 (38.1)	4 (36.4)	4 (40)	1
	Head/neck	13 (61.9)	7 (63.6)	6 (60)	
					
Received Treatment—no. (%)	Chemotherapy	16 (76.2)	7 (63.6)	8 (80)	0.64
	Radiation	19 (90.5)	10 (90.9)	9 (90)	1

**Table 2:** Baseline MFSI, SF-36, and POMS scores (mean ± SD) for cervical or head and neck cancer patients compared to normative data. Questionnaire scores/subscales that were significantly different after correction for multiple testing are indicated with an asterisk. Normative data for MFSI, SF-36, and POMS questionnaires from Cordero et al., 2012, Nyenhuis et al., 1999, and Ware et al., 1993, respectively. MFSI—Multidimensional Fatigue Symptom Inventory—Short Form, SF-36—RAND 36-Item Health Survey 1.0, POMS—Profile of Mood States—Short Form.

	Cancer Patients (n)	Normative Data (n)	*p*	*p* (adj)
MFSI General	8.5 ± 6.4 (21)	7.3 ± 5.9 (176)	0.413	0.966
MFSI Physical	5.0 ± 4.9 (21)	4.0 ± 4.4 (176)	0.390	0.966
MFSI Emotional	7.9 ± 6.0 (21)	4.0 ± 4.6 (176)	**0.009**	0.124
MFSI Mental	5.2 ± 4.8 (21)	4.2 ± 4.1 (176)	0.384	0.966
MFSI Vigor	9.7 ± 5.9 (21)	13.8 ± 5.6 (176)	**0.006**	0.101
SF-36 Physical Functioning *	55.5 ± 28.6 (19)	84.2 ± 23.3 (2474)	**<0.001**	**0.006**
SF-36 Limitations Physical *	25.0 ± 36.3 (19)	81.0 ± 34.0 (2474)	**<0.001**	**<0.001**
SF-36 Limitations Emotional	56.1 ± 49.8 (19)	81.3 ± 33.0 (2474)	**0.041**	0.343
SF-36 Energy-Fatigue	46.6 ± 22.7 (19)	60.9 ± 21.0 (2474)	**0.014**	0.152
SF-36 Emotional Well Being	66.5 ± 19.3 (19)	74.7 ± 18.1 (2474)	0.080	0.467
SF-36 Social Functioning *	56.6 ± 32.4 (19)	83.3 ± 22.7 (2474)	**0.002**	**0.031**
POMS Tension-Anxiety	11.0 ± 7.7 (19)	7.0 ± 5.5 (432)	**0.037**	0.391
POMS Depression	11.3 ± 12.8 (20)	7.1 ± 8.4 (432)	0.162	0.829
POMS Anger–Hostility	7.3 ± 9.2 (20)	6.6 ± 6.7 (432)	0.740	0.966
POMS Vigor *	11.9 ± 6.7 (20)	20.2 ± 6.2 (432)	**<0.001**	**<0.001**
POMS Fatigue	9.3 ± 7.6 (20)	7.3 ± 5.7 (432)	0.259	0.933
POMS Confusion	7.6 ± 5.5 (19)	5.2 ± 4.1 (432)	0.076	0.579
POMS Total Mood Disturbance	33.0 ± 41.2 (19)	12.7 ± 29.6 (432)	**0.047**	0.440

## Data Availability

The data presented in this study are available on request from the corresponding author.

## Supplementary Materials

The following supporting information can be downloaded at: https://www.mdpi.com/article/10.3390/curroncol29110658/s1, Figure S1: Overall survival of cervical or head and neck cancer patients for one year following 7 weeks of adjunct testosterone or placebo, Figure S2: Percent change (average ± SD) in amino acid and associated metabolite concentrations from baseline to post-treatment for cancer patients receiving testosterone or placebo, Table S1: Serum cytokine concentrations at baseline and post-treatment in cancer patients receiving testosterone or placebo, Table S2: Welch’s *t*-test results comparing the percent change of serum cytokine concentrations from baseline to post-treatment between placebo and testosterone groups, Table S3: Mixed effects models results assessing the effects of time and treatment on questionnaire scores for cancer patients receiving testosterone or placebo, Table S4: Serum amino acid and associated metabolite concentrations at baseline and post-treatment in cancer patients receiving testosterone or placebo, and Table S5: Welch’s *t*-test results comparing the percent change of serum amino acid and associated metabolite concentrations from baseline to post-treatment between placebo and testosterone groups.
