# Supplementary material for: Impact of Adjunct Testosterone on Cancer-Related Fatigue: An Ancillary Analysis from a Controlled Randomized Trial
Source: Curr Oncol. 2022 Nov 1;29(11):8340–56. doi: 10.3390/curroncol29110658 (PMC9689748; doi:10.3390/curroncol29110658)
Supplement: Supplementary file 1 [file curroncol-29-00658-s001.zip › curroncol-1967094-supplementary.pdf]

Supplementary Materials

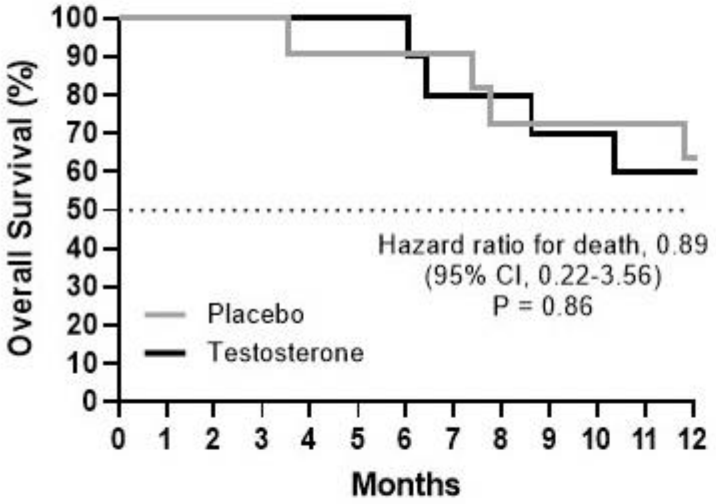

Figure S1. Overall survival of cervical or head and neck cancer patients for one year following 7 weeks of adjunct testosterone (n = 10) or placebo (n = 11). Survival at one year was not significantly different between the groups (P = 0.86); 1-year post-intervention survival was 63.6% in the placebo group and 60% in the testosterone group.

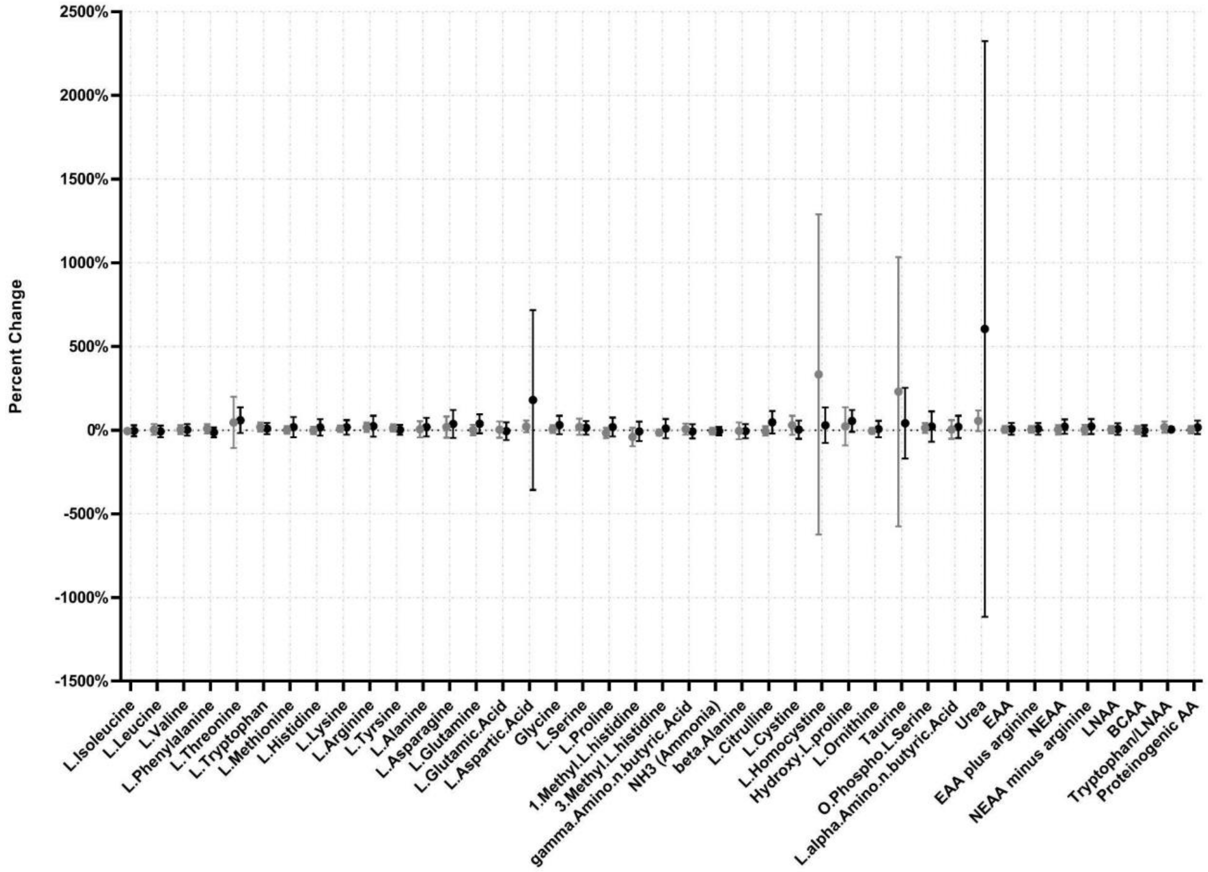

Figure S2. Percent change (average + SD) in amino acid and associated metabolite concentrations from baseline to post-treatment for cancer patients receiving testosterone (n = 9) or placebo (n = 9). There were no significant differences between groups after adjusting for multiple testing. Abbreviations: AA - amino acids, BCAA - branched chain amino acids, EAA - essential amino acids, LNAA - large neutral amino acids, NEAA- nonessential amino acids.

Table S1. Serum cytokine concentrations at baseline and post-treatment in cancer patients receiving testosterone or placebo. Abbreviations: GM-CSF - granulocyte-macrophage colony-stimulating factor , IFN-γ - interferon-gamma, IL - interleukin, tumor necrosis factor - TNF-α.

	**Placebo median (range, pg/mL, n = 11)**		**Testosterone median (range, pg/mL, n = 7)**	
	**Pre**	**Post**	**Pre**	**Post**
GM-CSF	53.4 (6.2-251.5)	39.9 (11.4-180)	111.7 (83.8-692.7)	105.5 (42.4-562)
IFN-γ	25.9 (2.6-49.1)	18.5 (3.5-43.9)	12.8 (8.5-31.8)	15.5 (4.2-25.6)
IL-1β	2.9 (0.6-7.2)	1.3 (0.2-7.4)	1.8 (1.5-3.2)	2.2 (0.7-3.3)
IL-2	3.1 (0.1-9.7)	1.7 (0.4-9.3)	3.6 (2.1-8.7)	1.7 (1.5-5.4)
IL-4	3.9 (0.6-16.8)	1.8 (0.6-83.9)	3.2 (0.6-7.3)	0.6 (0.6-7.9)
IL-5	2.4 (0.3-8.3)	2.4 (1.1-7.7)	4.7 (3-74.1)	4.5 (0.9-68.5)
IL-6	6.3 (1-57.3)	8.2 (2.2-20.6)	14.5 (4.3-153.2)	3.2 (2.7-102.8)
IL-7	7.5 (2.2-10.8)	7 (4.6-30.2)	8.6 (3-103.8)	6.8 (2.6-74.4)
IL-8	11.4 (3.7-33.2)	10.7 (5.7-23.4)	16.7 (6.7-32.8)	9.1 (7.9-19.1)
IL-10	10.2 (3.6-54.5)	8 (4.4-50.9)	11.8 (9.8-548.4)	10.5 (3.2-500)
IL-12	3.7 (0.4-8.2)	3.8 (0.1-7.9)	5.6 (3.6-249.9)	4.6 (2.3-149.4)
IL-13	2.9 (0-4.5)	3.1 (0.1-10.8)	5.4 (1.3-109.8)	3.6 (1.1-97.5)
TNF-α	12 (7-16.3)	9.9 (6.9-249.3)	15.6 (6.1-24.2)	11.7 (8-24)

Table S2. Welch's t-test results comparing the percent change of serum cytokine concentrations from baseline to post-treatment between placebo (n = 11) and testosterone groups (n=7). There were no significant differences between groups after adjusting for multiple testing. Abbreviations: GM-CSF - granulocyte-macrophage colony-stimulating factor , IFN-γ - interferon-gamma, IL - interleukin, tumor necrosis factor - TNF-α.

	**p**	**p (adj.)**	**Difference between means (placebo-testosterone, 95% CI)**
GM-CSF	0.069	0.508	0.567 (-0.051 to 1.185)
IFN-γ	**0.045**	0.397	0.515 (-0.013 to 1.017)
IL-1β	0.658	0.782	-0.112 (-0.648 to 0.425)
IL-2	0.195	0.697	0.838 (-0.500 to 2.175)
IL-4	0.533	0.782	0.425 (-1.00 to 1.853)
IL-5	0.161	0.697	0.869 (-0.4040 to 2.141)
IL-6	**0.010**	0.125	1.2 (0.341 to 2.067)
IL-7	0.122	0.647	0.469 (-0.142 to 1.081)
IL-8	0.276	0.725	0.258 (-0.228 to 0.744)
IL-10	0.157	0.697	0.221 (-0.096 to 0.147)
IL-12	0.077	0.514	0.749 (-0.095 to 1.592)
IL-13	**0.025**	0.258	1.543 (0.240 to 2.846)
TNF-α	0.307	0.725	1.676 (-1.790 to 5.142)

Table S3. Mixed effects models results assessing the effects of time and treatment on questionnaire scores for cancer patients receiving testosterone (n=10) or placebo (n=10 for VAS Fatigue and VAS Nausea, n=11 for all other scores). Uncorrected p-values are presented for the main effects of time point and treatment, and the interaction effect of time point by treatment for each questionnaire score/subscale. The ratio of missing values to total values is also presented. There were no significant main or interaction effects after correction for multiple testing. Abbreviations: BFI- Brief Fatigue Inventory, VAS - Visual Analog Scale, MDASI - M.D. Anderson Symptom Inventory, MFSI - Multidimensional Fatigue Symptom Inventory - Short Form, SF-36 - RAND 36-Item Health Survey 1.0, POMS - Profile of Mood States - Short Form.

Questionnaire/Subscale	Missing Values/Total Values (%)	Time point p-value	Treatment p-value	Time point x Treatment p-value	Difference between predicted means (Placebo - Testosterone, 95% CI)
BFI	31/168 (18.5%)	0.28	0.57	0.37	-0.5 (-2.4 to 1.4)
VAS Fatigue	30/168 (17.9%)	0.84	0.91	0.62	-0.1 (-2.3 to 2.1)
VAS Nausea	29/168 (17.3%)	0.52	0.60	0.96	0.5 (-0.9 to 1.4)
MDASI					
Interference	36/168 (21.4%)	0.19	0.65	0.71	-0.4 (-2.0 to 1.3)
Severity	30/168 (17.9%)	0.13	0.64	0.99	-0.3 (-1.6 to 1.0)
MFSI					
General	3/63 (4.8%)	0.27	0.30	0.45	-2.8 (-8.4 to 2.7)
Physical	3/63 (4.8%)	0.48	0.92	0.56	0.2 (-4.1 to 4.5)
Emotional	3/63 (4.8%)	0.51	0.46	0.63	-1.6 (-6.1 to 2.9)
Mental	3/63 (4.8%)	0.73	0.98	0.37	-0.1 (-4.2 to 4.0)
Vigor	3/63 (4.8%)	0.28	0.49	0.28	1.2 (-2.3 to 4.7)
Total	3/63 (4.8%)	0.62	0.54	0.50	-5.6 (-24.6 to 13.3)
RAND					
Physical Functioning	4/63 (6.4%)	0.27	0.60	0.49	5.8 (-16.8 to 28.3)
Energy/Fatigue	4/63 (6.4%)	0.51	0.72	0.49	2.6 (-12.0 to 17.2)
Emotional Well Being	4/63 (6.4%)	0.27	0.21	0.56	8.7 (-5.3 to 22.7)
Social Functioning	4/63 (6.4%)	0.86	0.33	0.72	9.1 (-9.8 to 27.9)
Pain	4/63 (6.4%)	0.34	0.97	0.16	0.3 (-18.5 to 19.1)
General Health	5/63 (7.9%)	0.65	0.56	0.71	4.8 (-12.2 to 21.7)
POMS					
Tension-Anxiety	7/63 (11.1%)	0.72	0.74	0.57	-0.9 (-6.6 to 4.8)
Depression	6/63 (9.5%)	0.90	0.95	0.97	-0.3 (-10.3 to 9.7)
Anger-Hostility	6/63 (9.5%)	0.65	0.90	0.29	0.4 (-6.5 to 7.4)
Vigor	7/63 (11.1%)	0.34	0.04	0.31	4.5 (0.2 to 8.8)
Fatigue	8/63 (12.7%)	0.37	0.35	0.18	-2.6 (-8.3 to 3.1)
Confusion	8/63 (12.7%)	0.36	0.80	0.76	-0.6 (-5.4 to 4.2)
Total Mood Disturbance	8/63 (12.7%)	0.72	0.64	0.63	-7.6 (-41.1 to 26.0)

Table S4. Serum amino acid and associated metabolite concentrations at baseline and post-treatment in cancer patients receiving testosterone or placebo. Abbreviations: AA - amino acids, BCAA - branched chain amino acids, EAA - essential amino acids, LNAA - large neutral amino acids, NEAA- nonessential amino acids.

	Placebo Median (Range, µM, n =9)		Testosterone Median (Range, µM, n = 9)	
	**Pre**	**Post**	**Pre**	**Post**
L-Isoleucine	45.1 (18.6-73.6)	33.8 (18-87.7)	41.3 (20.4-100.3)	39.6 (16.5-98.1)
L-Leucine	78.7 (53.9-135.6)	79.4 (40.2-179.6)	83 (47.7-196)	71.1 (44.5-148.8)
L-Valine	133.7 (90.9-239.9)	131.5 (74.7-305.7)	150.1 (75.8-307.4)	131.6 (93.4-276.9)
L-Phenylalanine	48 (30.4-80.3)	45.8 (39.7-90.4)	58 (25.4-111.9)	45.05 (19.7-71.9)
L-Threonine	73.4 (22.4-152.9)	83.4 (57.9-167.6)	64.2 (44.1-111.3)	101.3 (60-151.2)
L-Tryptophan	27.1 (13.6-50.9)	33.1 (19.6-43.9)	30.1 (20.2-67.3)	29.9 (24-60.4)
L-Methionine	12.3 (7.8-34.4)	14.6 (7.6-30.8)	15.2 (7.1-28.1)	13.2 (8.2-26.3)
L-Histidine	36.2 (25.6-65.5)	38.1 (25.6-59.4)	32.7 (18-90.5)	35.2 (27.2-69.1)
L-Lysine	93.9 (63.9-187.8)	112.1 (68-219.3)	99.5 (52-179.9)	116.6 (69.8-193.4)
L-Arginine	66.6 (43.5-109.2)	74.5 (61.4-90.5)	61.4 (35.5-125.2)	70.75 (60.6-108.8)
L-Tyrosine	31.2 (21.7-69.6)	35.2 (24.7-73.6)	40 (21.9-92.8)	36.9 (25.5-77.3)
L-Alanine	167.1 (101.1-399.2)	213.1 (119.6-354.5)	206.6 (104.2-395.2)	202.45 (145.6-329)
L-Asparagine	25.4 (12.2-76.7)	22.4 (18.1-96.6)	26.8 (13.6-115.7)	25.3 (1.5-112.4)
L-Glutamine	402.2 (312-575.2)	371.1 (308.4-706)	367.5 (185.8-621.5)	438.9 (233.4-752.9)
L-Glutamic acid	69.8 (25.1-109)	62.7 (25.5-131.7)	136.8 (50.8-169.9)	66.4 (41.7-274.9)
L-Aspartic acid	30.5 (12.3-45)	32.1 (20.9-48.4)	23.6 (2.6-49.8)	27.1 (16.6-44.4)
Glycine	219.7 (161.7-393.2)	215.9 (154-419.1)	160.6 (94.9-369.7)	201.65 (153.8-426.3)
L-Serine	110.6 (57.9-156.3)	101.3 (81.3-171.7)	91 (63.9-168.6)	105.45 (73.8-144.3)
L-Proline	70.2 (41.1-239.8)	49.9 (36.4-333.1)	73.2 (23.8-446.1)	57.05 (43.9-274.6)
1-Methyl-L-histidine	7.2 (1.4-32.5)	2.3 (0.8-27.1)	5.8 (2-47.4)	5.75 (1.3-23.3)
3-Methyl-L-histidine	3 (1.2-8.5)	2.6 (1.7-9)	2.3 (1.1-10.7)	2.5 (0.1-6.3)
gamma-Amino-n-butyric acid	110.1 (1.5-171.1)	101 (1.5-171.1)	92 (10.8-178.7)	86.65 (11.6-166.6)
NH3 (Ammonia)	72.7 (27.9-110.6)	68.8 (47.7-116.2)	95.7 (53.4-116.9)	68.9 (53.8-164.5)
beta-Alanine	47.6 (1.5-57.8)	34.9 (21.9-66.9)	52.1 (20.7-77.1)	54 (1.5-80.9)
L-Citrulline	20.7 (9.9-40.4)	20.4 (8.6-56.6)	17.2 (9.6-55.7)	21.45 (10.5-39.7)
L-Cystine	20.1 (3.9-50.6)	21.8 (9-60.3)	24 (9.7-75.5)	23.4 (1.5-73.1)
L-Homocystine	4.1 (0.3-7.2)	4 (0.4-13.4)	2.9 (1.3-4.2)	3.55 (0.4-15.6)
Hydroxy-L-proline	5.6 (1.6-19.4)	6.4 (1.7-31)	4 (1.8-25.8)	5.6 (1.4-25.4)
L-Ornithine	35.6 (30.2-137.1)	37.9 (23.1-111.1)	50.5 (32.5-119.1)	46.45 (30.2-91.9)
Taurine	125.9 (1.5-167.2)	62.9 (32.7-110.5)	135.9 (1.3-171.5)	45.5 (9-115.2)
O-Phospho-L-Serine	6.2 (4.2-8.5)	6.9 (5.6-8.4)	5.9 (3.1-13.4)	6.4 (5-11.9)
L-alpha-Amino-n-butyric acid	13.7 (7-43.8)	12.5 (3.3-54)	10.1 (4.6-33.3)	10.95 (4-57.8)
Urea	2346.5 (862.8-5609.1)	3150.4 (1531.9-7045)	2175 (145.8-7300.2)	3069.3 (1157-7704)
EAA	531.2 (395.2-1004.5)	581.4 (361-1175)	617 (318.1-1192.7)	566.65 (455.1-1060.6)
EAA (+arginine)	595.3 (443.3-1048)	657.2 (426.7-1249.5)	652.5 (379.5-1317.9)	633.15 (526.8-1159.6)
NEAA	1241.9 (877.2-2031.1)	1219.2 (972-2225)	1154 (651.3-2310.7)	1321.35 (1042.6-2222.7)
NEAA (-arginine)	1169.9 (829.1-1987.6)	1153.6 (902-2141.2)	1118.5 (589.9-2185.5)	1252 (972.9-2113.9)
LNAA	479.8 (341.8-874.4)	500.1 (317.7-1029.3)	568.9 (288-1105.6)	488.8 (415.6-936.4)
BCAA	253.7 (166.6-443.5)	243.9 (138.6-573)	260.2 (143.9-603.7)	234.25 (164.9-523.8)
Tryptophan/LNAA ratio	0.06 (0.04-0.07)	0.06 (0.04-0.07)	0.06 (0.05-0.07)	0.06 (0.05-0.08)
Proteinogenic AA	1812 (1272.4-3035.6)	1815 (1360.7-3294.5)	1771 (969.4-3503.4)	1900.15 (1561.2-3197.2)

Table S5. Welch's t-test results comparing the percent change of serum amino acid and associated metabolite concentrations from baseline to post-treatment between placebo (n=9) and testosterone (n=9) groups. Uncorrected p-values are shown. There were no significant differences between groups after correcting for multiple testing. Abbreviations: AA - amino acids, BCAA - branched chain amino acids, EAA - essential amino acids, LNAA - large neutral amino acids, NEAA- nonessential amino acids.

	**p**
L-Isoleucine	0.84
L-Leucine	0.50
L-Valine	0.95
L-Phenylalanine	0.13
L-Threonine	0.82
L-Tryptophan	0.52
L-Methionine	0.43
L-Histidine	0.38
L-Lysine	0.61
L-Arginine	0.77
L-Tyrosine	0.40
L-Alanine	0.60
L-Asparagine	0.60
L-Glutamine	0.11
L-Glutamic acid	0.70
L-Aspartic acid	0.40
Glycine	0.24
L-Serine	0.79
L-Proline	0.13
1-Methyl-L-histidine	0.23
3-Methyl-L-histidine	0.28
gamma-Amino-n-butyric acid	0.43
NH3 (Ammonia)	0.98
beta-Alanine	0.94
L-Citrulline	0.05
L-Cystine	0.33
L-Homocystine	0.37
Hydroxy-L-proline	0.48
L-Ornithine	0.53
Taurine	0.51
O-Phospho-L-Serine	0.79
L-alpha-Amino-n-butyric acid	0.62
Urea	0.37
EAA	0.77
EAA (+arginine)	0.79
NEAA	0.31
NEAA (-arginine)	0.30
LNAA	0.85
BCAA	0.83
Tryptophan/LNAA ratio	0.26
Proteinogenic AA	0.39
